# Editorial: Functional Nanomaterials for Sensor Applications

**DOI:** 10.3390/nano12213750

**Published:** 2022-10-25

**Authors:** Noel Rodriguez, Diego P. Morales, Almudena Rivadeneyra

**Affiliations:** Department of Electronics and Computer Technology, University of Granada, 18071 Granada, Spain

Functional nanomaterials have become one of the most fascinating fields in nanotechnology. The notable interest that they inspire relies on the fact that these nanomaterials constitute the driving force for advanced research in many fields, including nanosized energy conversion, environmental sustainability, catalysts, electronic devices, pervasive sensors, biomedical engineering, and more. This genuine interest is undoubtedly motivated by their unique structure and properties paired with a massive potential for integration in industrial applications.

This Special Issue aims at offering readers a compilation of cutting-edge research regarding the synthesis, development, characterization and utilization of functional nanomaterials, covering a wide spectrum of technologies and applications, serving as a guide for new students of the field as well as established researchers.

This Special Issue is nourished with a variety of topics dealing with carbon-based functional materials [1,2,3,4] exploring different transduction mechanisms as well as other emerging technologies with relevance in the biology field [5,6,7,8,9]. Characterization techniques [10] and a comprehensive review on wearable optical sensors under different powering approaches [11] are covered.

The results and findings are expected to be useful for researchers who are working in the field of functional nanomaterials. Finally, the editors would like to express their sincere gratitude to all authors who contributed their innovative research to this Special Issue.
